# Ecological Routes of Avian Influenza Virus Transmission to a Common Mesopredator: An Experimental Evaluation of Alternatives

**DOI:** 10.1371/journal.pone.0102964

**Published:** 2014-08-15

**Authors:** J. Jeffrey Root, Kevin T. Bentler, Susan A. Shriner, Nicole L. Mooers, Kaci K. VanDalen, Heather J. Sullivan, Alan B. Franklin

**Affiliations:** United States Department of Agriculture, National Wildlife Research Center, Fort Collins, CO, United States of America; Kobe University, Japan

## Abstract

**Background:**

Wild raccoons have been shown to be naturally exposed to avian influenza viruses (AIV). However, the mechanisms associated with these natural exposures are not well-understood.

**Methodology/Principal Findings:**

We experimentally tested three alternative routes (water, eggs, and scavenged waterfowl carcasses) of AIV transmission that may explain how raccoons in the wild are exposed to AIV. Raccoons were exposed to 1) water and 2) eggs spiked with an AIV (H4N6), as well as 3) mallard carcasses experimentally inoculated with the same virus. Three of four raccoons exposed to the high dose water treatment yielded apparent nasal shedding of >10^2.0^ PCR EID_50_ equivalent/mL. Little to no shedding was observed from the fecal route. The only animals yielding evidence of serologic activity during the study period were three animals associated with the high dose water treatment.

**Conclusions/Significance:**

Overall, our results indicate that virus-laden water could provide a natural exposure route of AIV for raccoons and possibly other mammals associated with aquatic environments. However, this association appears to be related to AIV concentration in the water, which would constitute an infective dose. In addition, strong evidence of infection was only detected in three of four animals exposed to a high dose (e.g., 10^5.0^ EID_50_/mL) of AIV in water. As such, water-borne transmission to raccoons may require repeated exposures to water with high concentrations of virus.

## Introduction

Although much of the research associated with influenza A viruses has been focused on avian species, the potential role of wild mammals in the ecology of these viruses has received attention for only a limited number of species [1]–[3]. For example, raccoons (*Procyon lotor*) have been found exposed to AIVs in certain locations in the U.S. and elsewhere [1], [4]; however, the mechanism(s) associated with the exposures of raccoons to AIVs are unclear.

Overall, the precise route of exposure of mammals to avian influenza viruses (AIV) is not well-understood [5]. It has been suggested that cross-species transmission of AIV to mammals may occur via physical contact between mammals and avian reservoirs (e.g., through predation and scavenging), indirect contact with excreta from birds or virus-contaminated environments (e.g., through ingestion of contaminated water), or through aerosols [5]. However, some of these scenarios might be more likely for highly pathogenic (HP) AIV when compared to low pathogenic (LP) AIV [5]. LP AIV infections are typically thought to be more localized within individuals compared to those of HP AIV which are thought to be more widely disseminated throughout the bodies of infected animals (see [6] and citations therein). Recent work on the persistence of a HP AIV in chicken carcasses suggested that infected muscle tissue could potentially deliver infectious virus for up to three days post-mortem and certain other tissues two to three days longer, depending on temperature [7]. Thus, the potential of LP AIV infections in wild mammals from scavenging on infected avian carcasses has not been thoroughly addressed.

Water is thought to be an important aspect in the natural transmission of avian influenza viruses [8], [9], as modeling efforts have suggested that some AIVs can survive for long time periods in water within certain temperature ranges [10]. For example, multiple viruses have been isolated from lake water in Alaska, with viruses detected at titers up to 10^2.8^ EID_50_ per mL of water [11] and isolates have been obtained from environmental samples from wetlands during the summer in California [12]. In addition, researchers recently noted relatively high titers (e.g., >10^5.0^ pfu/mL for one subtype) of virus accumulating in small pools of water in an experiment using mallards inoculated with AIV as the viral source [13]. In a study on water transmission of a LP AIV to mallards, approximately 10^3.0^ PCR EID_50_ equivalent/mL was sufficient to infect naïve ducks [14]. While Achenbach and Bowen [13] suggested that the viral titers they observed (up to >10^5.0^ pfu/mL of a LP H7N3 virus) in water might be extreme as compared to natural conditions, these titers may be plausible for LP AIVs in small or shallow water bodies. Raccoons are well-known to eat a diversity of foods which are often associated with water [15]. Therefore, this species could be more readily exposed to virus-contaminated water, as compared to species with diets that are less frequently associated with aquatic environments.

Although many experimental infections of AIV have been conducted on diverse species, very few studies [13], [14], [16] have addressed the natural modes of transmission of these viruses in experimental settings, which are fundamentally difficult questions to address. Thus, the objective of this study was to assess the transmission potential of a common AIV subtype (H4N6) of wild birds to raccoons from water and food sources that would be naturally used by this species.

## Methods

### Ethics statement

Animal experiments were approved by the Institutional Animal Care and Use Committee of the National Wildlife Research Center (NWRC), Fort Collins, CO, USA (Approval number 1810). All raccoons used in the studies listed below were born in captivity. The mallards used in this study were obtained from in-house sources at NWRC, and were initially purchased from a private breeder.

### Transmission of AIV through water

Ten raccoons were randomly assigned to one of two AIV doses or as control animals. Nine of these (8 treatment and one control) animals were housed in separate 2.4×1.2 m cages in the same animal room for experimental infections, while an additional control animal was housed in a different building to account for the potential of aerosol transmission to the control animal housed with treatment animals. All animals were supplied with a den box, a water bowl, a food bowl, a litter box, an enrichment toy, and a plastic water pan (45.7×32.4 ¾ cm) for virus introduction. The water containers, litter boxes, and food bowls were secured to the cages so their contents could not be readily discarded.

On day 0 of this experiment, all animals were anesthetized with a 5∶1 ratio of ketamine/xylazine; blood and a nasal swab were collected from each individual. Subsequently, water pans were spiked with an avian influenza virus (A/Mallard/CO/P70F1-03/08(H4N6)) that was originally isolated from a North American wild bird and passaged in a mallard during an experimental infection study and successively passaged through specific pathogen-free (SPF) embryonated chicken eggs. Treatments included water spiked to 10^5.0^ EID_50_/mL (high dose) and 10^3.1^ EID_50_/mL (low dose) as assessed by virus isolation in embryonated chicken eggs. The water pans were not disturbed by researchers until the pans were removed on 7 DPI.

The high dose was used to produce an extreme environmental condition [13] while the low dose was used to mimic an approximate dose of mallard shedding in a small water source [14]. Each water pan was filled with 3.785 L (2 gallons) of distilled water and virus stock was mixed into the water with a plastic stir stick. A different stir stick was used for each cage. The water pans were the sole source of water for the raccoons until 1 DPI, when an additional bowl of fresh water was added to each pen. Food (Omnivore Zoo Diet A, Mazuri Exotic Feed, Lincoln, NE) and fresh water (in water bowls only) were replaced daily. The spiked water in water pans was not replaced or refilled until 7 DPI, after which these pans were removed for the remainder of the experiment.

For daily sampling from 1–8 DPI, raccoons were anesthetized with isoflurane using custom built gas anesthesia chambers [17]. Daily processing consisted of nasal swabs (0–8 DPI), oral swabs (1–8 DPI), rectal swabs (1–8 DPI), fecal swabs (1–8 DPI, when available), and a water sample (0–7 DPI). All swabs were stored in 1 mL of BA-1 viral transport medium {see [18]}. Water pans were mixed briefly with a transfer pipette prior to sample collection. Samples were stored on ice packs until transfer to −80°C immediately following the conclusion of daily processing. All swab types were analyzed for animals that yielded nasal shedding, while roughly one-half of the non-nasal swabs were assayed for animals that did not yield nasal shedding (e.g., one-half of the animals tested on even days and the other one-half tested on odd days). Raccoons were again processed (i.e., swab collection) and bled on 14 DPI. Blood samples were placed in serum separator tubes, allowed to clot, and centrifuged. Serum was stored in cryovials at −80°C for serologic analyses. Subsequently, all animals were humanely euthanized on 15 DPI.

### Transmission of AIV through chicken eggs

The animal protocol of this experiment was essentially the same as the water experiment, with a few exceptions. Daily processing was conducted during 1–7 DPI and on 14 DPI when the animals were euthanized. A pan with an SPF embryonated chicken egg glued to the bottom was placed in each cage to help ensure the egg contents would not fall through the floor grate. In addition, a total of nine animals (8 treatment and 1 control) were housed in separate cages in the same animal room.

The SPF embryonated chicken eggs were spiked with an AIV by two methods based on previous observations of internal and external viral contamination of turkey eggs [19]. First, we spiked egg albumen to approximately 10^6.67^ EID_50_ of the same virus listed above (n = 4). Second, the shells of additional eggs were coated with mallard fecal matter spiked with the same virus. Two grams of fecal material was spiked to titers of approximately 10^5.0^ EID_50_/gram (n = 2) and 10^4.3^ EID_50_/gram (n = 2). These doses were based on viral shedding from mallards following exposures to contaminated water and mallard shedding following experimental infections [20]. Each raccoon received only one egg.

### Transmission of AIV through mallard carcasses

The animal protocol of this experiment was essentially the same as the water experiment, with the following exceptions. Daily processing was conducted during 1–8 DPI and 14 DPI when the raccoons were euthanized. Water pans were not included in the pens. A total of six raccoons were utilized in this experiment (5 treatment; 1 control).

Five mallards were orally inoculated with approximately 10^6.0^ EID_50_ of the same virus described above diluted into 1 mL of BA-1 viral transport media and were group housed in a single animal room. One mallard, which was held in a different room, was mock-inoculated with 1 mL of BA-1 containing no virus. Fecal and cloacal swabs were collected each day and immediately following euthanasia on 2 DPI. The mallard carcasses were offered to raccoons immediately following euthanasia.

### Laboratory testing

Swab samples were tested by real-time reverse-transcription polymerase chain reaction (RRT-PCR) for viral RNA detection and quantification. In general, previously described primers and methods were employed [21], [22], which have been described in detail elsewhere [23]. As in earlier studies, positive samples were defined as those yielding a two-well positive amplification with a Ct value of ≤38 and suspect positive samples were defined as those yielding a two-well positive amplification with a Ct value of >38 [23]. Virus isolation was conducted on key samples (e.g., positive nasal swabs and daily water samples from a water pan) in embryonated chicken eggs following published protocols [24].

Serum samples were analyzed with the FlockCheck Avian Influenza MultiS-Screen Antibody Test Kit (IDEXX Laboratories, Inc., Westbrook, ME). Although this test has been evaluated for several experimentally infected wild bird species [25], naturally infected wild bird species [26], and swine [27], its applicability for use in other species has not been thoroughly examined. Therefore, we did not use a stringent cutoff threshold to assess serological activity in raccoons. Rather, we evaluated the differences in sample-to-negative (S/N) ratios from pre- and post-experiment serum samples. Serological activity was implied when changes in S/N ratios were multi-fold greater than those of control animals (see results).

## Results

### AIV shedding in raccoons infected from water

Because we were not directly infecting raccoons with AIV, this experiment relied on raccoons using their water pans for various activities (e.g., drinking, manipulating food, etc.). At 1 DPI, it was obvious through food discoloration present in water pans that six of eight treatment animals had used their water pans. By 2 DPI, all animals had obviously used their water pans. Although two of the water pans still had approximately one-half of their initial volume in them when they were removed, most of the others had less than one-fourth of their initial volume, and two were nearly empty. We tested for virus longevity from the water pan of a high dose animal. Virus isolation indicated positive results from each day tested (0–7 DPI).

Nasal swabs positive for viral RNA were detected from the high dose treatment group, but none of the low dose treatment animals nor the control animals yielded any RNA positive nasal swabs. The highest EID_50_ equivalent/mL from a nasal swab was detected from a high dose animal during 5 DPI, yielding 10^4.2^ PCR EID_50_ equivalents/mL (**Table 1**). However, most nasal swabs yielding positive results were lower in apparent quantity than the sample mentioned above (**Table 1**). No animal yielded evidence of nasal shedding at 14 DPI. All tested (Ct <38) nasal swabs were positive for live virus (**Table 1**).

**Table 1:** Nasal shedding (as log_10_ PCR EID_50_ equivalents/mL) of an avian influenza virus by raccoons infected through contact with virus-laden water (high dose treatment).

Animal	0 DPI	1 DPI	2 DPI	3 DPI	4 DPI	5 DPI	6 DPI	7 DPI	8 DPI	14 DPI
A	–a	–	3.4*	2.3*	2.1*	4.2*^b^	2.7*	–	S	–
B	–	S	–	–	–	–	2.6*	S	S	–
C	–	–	S	–	3.2*	S	–	S	–	–
D	–	–	–	–	–	–	–	–	–	–

a Nasal shedding was assessed via nasal swabs by RRT-PCR. A dash “—” indicates that no viral RNA was detected. An asterisk “*” indicates that live virus was confirmed in sample by virus isolation. Virus isolation was only attempted with samples with Ct values <38.

b Because initial results were much higher than most other samples, a second RRT-PCR run was conducted. Results presented represent the mean among the two independent runs.

The subset of oral, fecal, and rectal swabs tested yielded some positive or suspect positive results by RRT-PCR. However, most were typically low in apparent quantities. For example, positive oral swabs were detected from individuals from the high dose treatment (individuals A and C on 2, 4, and 5 DPI and 2, 3, 4, and 6 DPI, respectively). The highest PCR EID_50_ equivalent detected was 10^3.2^/mL on 2 DPI. Fecal swabs also yielded some low quantity positive results for this high dose treatment group. However, all of the samples had Ct values near our threshold of 38, and the only positive results (Ct <38) that were obtained were from animal D, which did not yield strong evidence of nasal shedding. In addition, positive fecal results were not obtained from this animal after 7 DPI (when the water pans were removed), thereby suggesting that the positive results we observed were associated with direct contamination (e.g., water splashes) from the spiked water pan and were not representative of fecal shedding. This is further supported by our observation that no rectal swabs met our criteria for a positive sample, although some from the high dose water treatment group, including the animal mentioned above, were considered as suspect positive. Due to the lack of nasal shedding, roughly one-half of other swab types of the low dose treatment and control animals were tested each DPI. None yielded positive results.

### AIV shedding in raccoons infected from eggs

Several eggs were consumed within a few hours following the introduction of eggs into raccoon pens. During the same time-frame, one raccoon apparently (e.g., this was not observed but inferred) licked the feces off of the egg it was offered without breaking the egg, and consumed the egg approximately 2.5 hours later. By 2 DPI all eggs were consumed and the egg pans were removed. While some animals ate the vast majority of their eggs with little evidence of spillage, others produced spillage of yolk and pieces of shell on the floor of the animal room. Therefore, it is clear that some of these animals did not consume the entire egg they were offered.

Regardless of the amount of egg consumed, no viral RNA was detected from nasal swabs from any raccoon during any DPI sampled. Roughly one-half of other swab types were tested each DPI (e.g., one-half of the animals each DPI) and none yielded positive results.

### AIV shedding in raccoons scavenging infected waterfowl carcasses

All inoculated mallards subsequently used for carcasses yielded evidence of viral shedding by 2 DPI of the experiment, although results were variable among individuals. For example, although the cloacal swabs from one treatment mallard only reached suspect positive levels (i.e., slightly above the 38 Ct cutoff), others reached >10^2.0^ (n = 2), >10^3.0^, and, in one instance, approximately 10^5.0^ PCR EID_50_ equivalent/mL. In addition, a mallard fecal sample reached >10^5.0^ PCR EID_50_ equivalent/mL during 2 DPI. Thus, the infection levels in the mallards used for carcasses likely represented a range of infection levels from low to moderately high. The control mallard yielded no evidence of viral shedding at 2 DPI or earlier. Carcass consumption varied by individual raccoon. For example, one raccoon consumed nearly an entire mallard with the exception of the mallard's spinal cord and wings by 1 DPI. Two other carcasses were approximately one-half consumed by 1 and 2 DPI, respectively. By 3 DPI these carcasses were essentially completely consumed. Remaining carcasses and/or bird parts were removed at 4 DPI.

Although several raccoons nearly completely consumed the mallard carcasses that were shedding AIV RNA prior to euthanasia, none yielded any evidence of nasal shedding during 1–8 DPI. Due to the lack of evidence of nasal shedding by raccoons, roughly one-half (i.e., one-half of the animals each DPI) of other swab types were tested each DPI. None yielded positive results and a single animal yielded a suspect positive oral swab on 3 DPI.

### Serology in raccoons

Three of the four animals in the high dose water treatment yielded evidence of serologic activity by 14 DPI of the water experiment, as their S/N ratios drastically decreased by the end of the experiment (**Table 2**). A fourth animal in the high dose water treatment did not show evidence of serologic activity (**Table 2**). However, it is unclear if antibody positive animals in this group are a result of repetitive exposure to AIV as compared to a productive infection. Control animals and those associated with the low dose treatment yielded no evidence of serologic activity from serum samples collected at the end of the experiment (**Table 2**).

**Table 2:** Antibody assessments in raccoons experimentally infected with an avian influenza virus through contact with virus-laden water (high dose treatment).

Animal	0 DPI	14 DPI	Change in S/N ratio^a^	Probable Serologic response
A	0.88	0.25	−0.63	Yes
B	0.83	0.50	−0.33	Yes
C	0.83	0.38	−0.45	Yes
D	0.88	0.85	−0.03	No

a Numerical values represent sample-to-negative ratios from pre- and post-experiment serum samples.

No animals yielded evidence of seroconversion following the egg experiment, as the S/N ratios increased in all but one animal when pre-experiment serum and post-experiment serum were compared. Following the mallard carcass experiment, the S/N ratios decreased in some animals when 14 DPI and pre-experiment serum samples were compared. However, the largest decrease noted in a treatment animal was similar to that of the control animal, thereby suggesting that the observed minor change was not associated with a serological response.

## Discussion

Raccoons are known to be exposed to AIV and antibodies to these viruses have been detected in raccoons from multiple areas in the U.S. [1] and from introduced populations in Japan [4]. The mechanisms by which these mesopredators are naturally exposed to these viruses have not been thoroughly evaluated. Hall et al. (2008) proposed that high antibody prevalence among raccoons in select states could be a function of localized concentrations of both raccoons and waterfowl in landscapes with limited riparian areas [1]. Recent research has indicated that artificial water bodies containing approximately 10^3.0^ PCR EID_50_ equivalents/mL were sufficient to cause infections in mallards [14]. Considering that water is thought to be an important facet in the “traditional” transmission of AIVs [8], virus-contaminated water might also be an important mechanism for the transmission of these viruses to species not traditionally associated with their epidemiology, such as raccoons and certain other mammal species associated with aquatic environments. Results from the present study suggest that transmission of one common AIV subtype to raccoons from water is possible; however, this exposure route may be limited to relatively high concentrations of virus in natural water sources or possibly repeated exposure events. Furthermore, contamination of the nasal cavity from virus laden water cannot be ruled out as a possible scenario for the apparent nasal shedding we observed.

Nasal shedding was the most prominent route of shedding during this study. Three of four of the animals in the high dose water treatment group yielded evidence of nasal shedding. Of interest, the single animal of this treatment group that did not show clear evidence of nasal shedding during this study was observed to have a fairly clean water pan until 2 DPI. The three other animals in this group had used their water pans by 1 DPI. It is unclear if this behavior affected subsequent shedding or apparent contamination of the nasal cavity.

Other swab types (e.g., oral and fecal) occasionally yielded positive RRT-PCR results, although many of these were at or near the threshold of detection. It should be noted that a spiked water source was present in the pens from 0–7 DPI; therefore, contamination of feces from this viral source and residual virus in the oral cavity from drinking from the water pans cannot be ruled out as the reason for these positive RRT-PCR results. However, rectal swabs should have had reduced potential contamination issues when compared to the oral and fecal swabs collected in this study. Nonetheless, none yielded clear positive results, although three individuals yielded suspect positive results. Therefore, these data lend little support to large quantities of viral shedding in feces by raccoons. Although some influenza A viruses can replicate in the intestinal tissues of some mammal species [28], Hall and others [1] noted a very small quantity of viral RNA on a single rectal swab following experimental infections of raccoons with a human H3N2 influenza A virus.

The frequency which raccoons “wash” their food is debatable [29], and some have argued that “dousing” may be a more appropriate term for this behavior [30]. In addition, differences in this type of behavior among captive versus wild-caught raccoons have been suggested [31]. The obvious discoloration of water, along with direct observations of raccoons dipping their food in water, suggest that the raccoons studied in this project wash and otherwise manipulate their food in water with high frequency. In addition, some of the water pans used in this study were nearly empty by 7 DPI while others retained approximately one-half of their volume, thereby suggesting that select individuals drank, splashed, played in, or otherwise utilized their water pans frequently. Subsequently, these individuals were likely exposed to virus-laden water on multiple occasions.

Three of four animals from the high viral dose group in the water experiment yielded evidence of serologic activity by 14 DPI (**Table 2**). On average, the change in S/N ratios of these animals was 0.47 when comparing serum samples collected at 0 and 14 DPI. The final animal in the high spiked water treatment group, which did not yield clear evidence of infection, yielded no evidence of serological activity by 14 DPI.

The occurrence of wild mammals on poultry production facilities has been thought to be a risk factor associated with the spread of a LP AIV among these facilities [32]. Although we were able to detect live virus in all raccoon nasal swab samples that were tested, the limited viral RNA shed by most raccoons during the present study suggests that their role, if any, in the spread of LP AIV is likely limited. However, we did observe individual variation in shedding quantities, with one of four individuals in the high-dose water experiment shedding more consistently and in higher quantities than the others.

It is undetermined why LP AIV infections in mammalian wildlife that prey or scavenge on wild waterfowl and poultry are not commonly observed or reported [5]. We attempted to address this question in one of our studies. However, duck carcasses fed to raccoons failed to yield any evidence of having caused infections in raccoons with the LP AIV we used. Of interest, clinical signs of disease, mortality, and gross and histological lesions were observed in red-legged partridge (*Alectoris rufa*) experimentally infected with HP AIV H7N1 virus while only moderate shedding and seroconversions were observed in some individuals of this species infected with a LP H7N9 virus [33], thereby suggesting that the highly pathogenic form of AIV produced more virus in these birds. LP AIV infections are typically thought to be more limited to the respiratory and/or gastrointestinal tracts when compared to those of HP AIV which can cause disseminated infections (reviewed by [6]). Thus, for obvious reasons, transmission of AIVs through predation or scavenging appears to be much more likely for HP as compared to LP AIVs. We propose that the limited distribution of virus that likely occurred in the mallards we infected was key to the lack of positive results we observed in raccoons. However, LP AIV infected carcasses could potentially lead to infections in natural settings if more virus is present in ingested materials.

Virus-contaminated eggs also failed to produce infections in raccoons. In a comprehensive review, it was suggested that occurrences of LP AIV in the internal contents of eggs was absent or rare, but fecal contamination of shells was possible [6]. However, LP AIV was recently detected in the internal contents of and on the shell of turkey eggs [19], which served as the motivation for our examination of AIV transmission through eggs. Regardless, either the virus and doses we used in the present study were not effective in causing infections in raccoons fed virus-spiked eggs or the oral route of inoculation is not effective or efficient in this species. The potential for this type of transmission, however, should not be completely discounted. As waterfowl nest predators [34], raccoons may consume entire egg clutches during foraging events, rather than just a single egg. If eggs were contaminated either internally or externally, consuming a large quantity of eggs at once could produce the requisite viral dose for infection. In addition, it is possible that our positive water experiment results were augmented by inadvertent nasal inoculations (i.e., virus laden water splashed in a nostril while drinking), whereas the egg and carcass debris were less likely to have been drawn into the nasal cavities.

Virus contaminated water is thought to be a major route of infection in the natural transmission cycle of AIV in waterfowl populations [8], [14]. Overall, it is not well-established how other species, especially those not thought to be traditionally involved in epidemiology of AIVs, might be affected by this natural transmission cycle. Of interest, cross-species transmission of a LP H7N3 virus to blackbirds, pigeons, and rats exposed to infected mallards, their excrement, and virus-contaminated water, as compared to a similar experiment associated with the transmission of a LP H5N2 virus to the same species, was thought to have potentially been more successful due, to some degree, to higher titers of virus deposited in the shared water source associated with the former virus [13]. The present study broadens the potential importance of virus-laden water to the transmission of AIV to a common wild mesocarnivore, and lends additional support to previous studies indicating that this type of transmission may be enhanced by larger viral loads in water sources.
